# Supplementary material for: PIM kinase control of CD8 T cell protein synthesis and cell trafficking
Source: eLife. 2025 May 13;13:RP98622. doi: 10.7554/eLife.98622 (PMC12074636; doi:10.7554/eLife.98622)
Supplement: Figure 2—source data 1. [file elife-98622-fig2-data1.zip › Figure 2 - Source Data 1/Fig_2A_labelled_blot_PIM2.pdf]

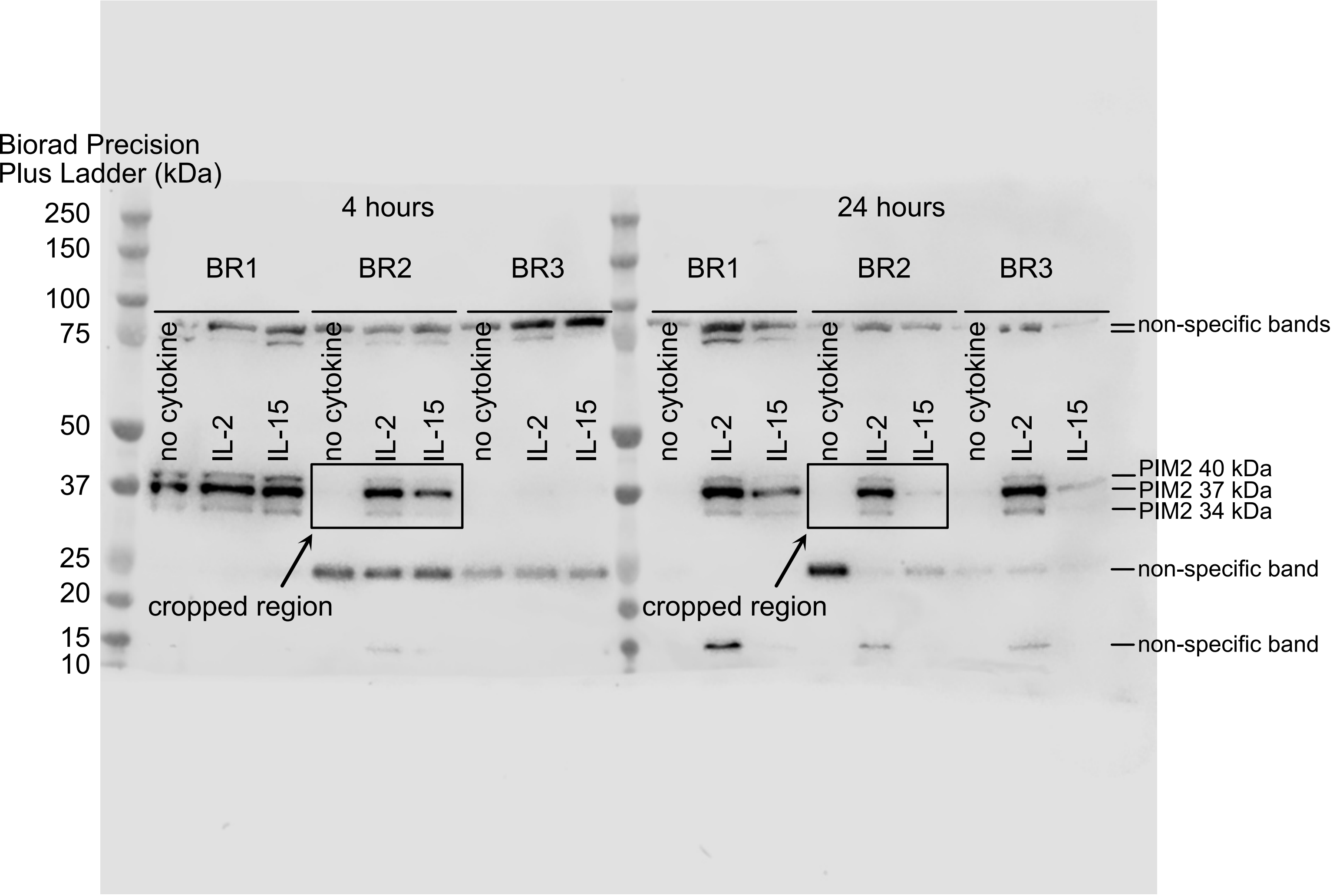

Figure 2 - source data 1

Uncropped and labelled membrane corresponding to Figure 2, panel A,
