# Supplementary figures and images for: PIM kinase control of CD8 T cell protein synthesis and cell trafficking
Source: eLife. 2025 May 13;13:RP98622. doi: 10.7554/eLife.98622 (PMC12074636; doi:10.7554/eLife.98622)

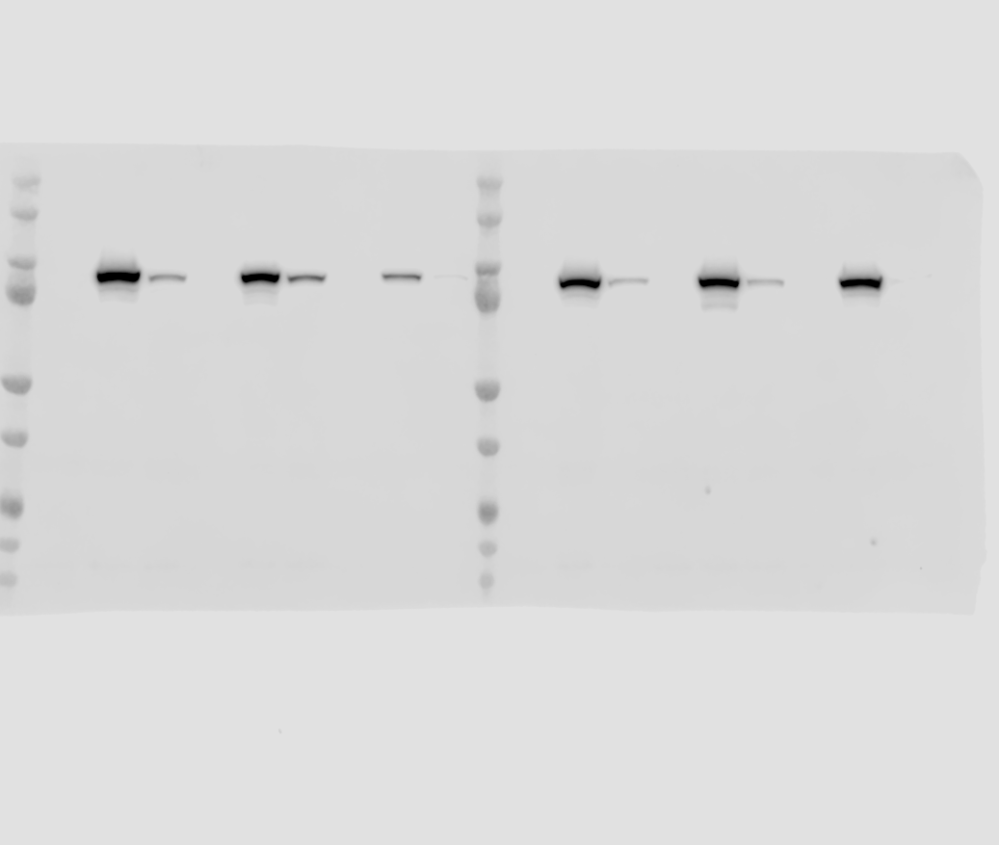

Supplement: Figure 2—source data 2. [file elife-98622-fig2-data2.zip › Figure 2 - Source Data 2/Fig_2A_raw_blot_STAT5_Y694_on_PIM1_membrane.png]

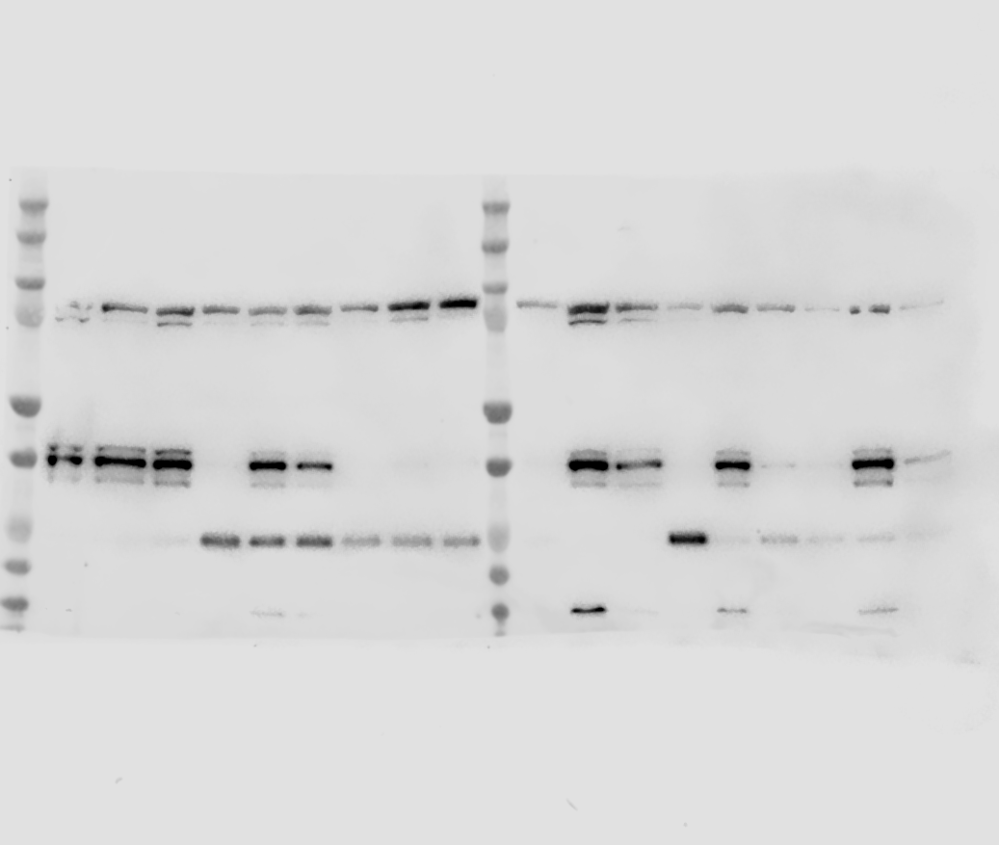

Supplement: Figure 2—source data 2. [file elife-98622-fig2-data2.zip › Figure 2 - Source Data 2/Fig_2A_raw_blot_PIM2.png]

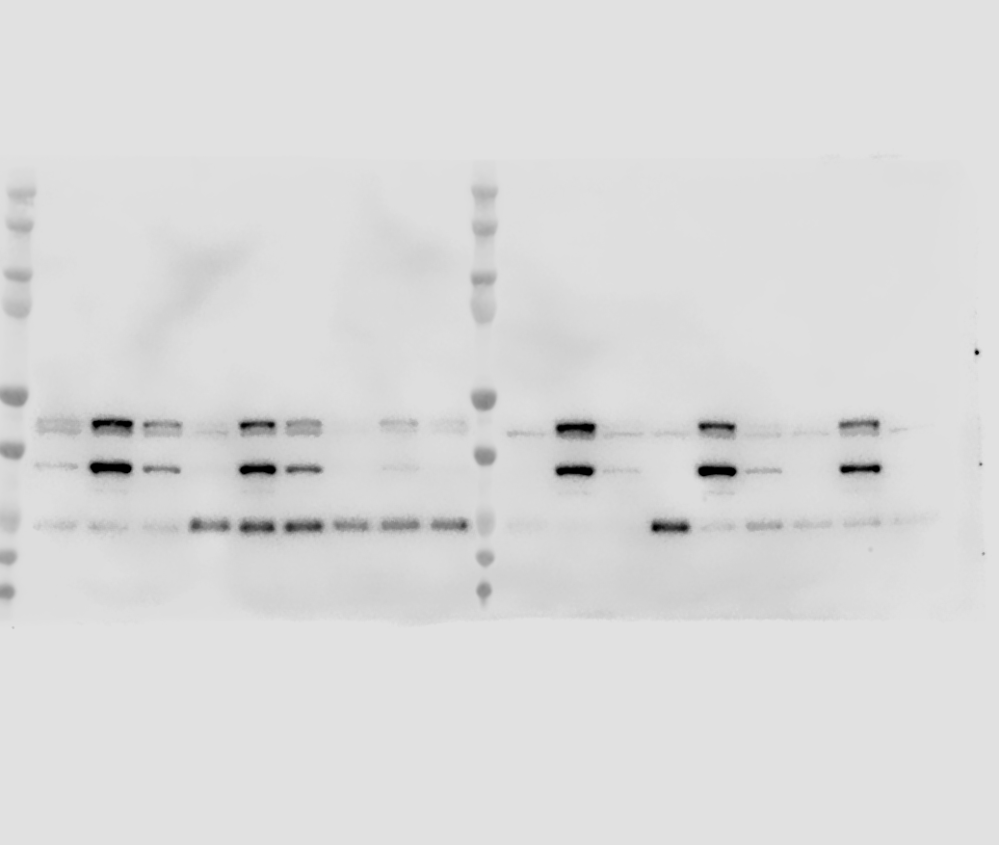

Supplement: Figure 2—source data 2. [file elife-98622-fig2-data2.zip › Figure 2 - Source Data 2/Fig_2A_raw_blot_PIM1.png]

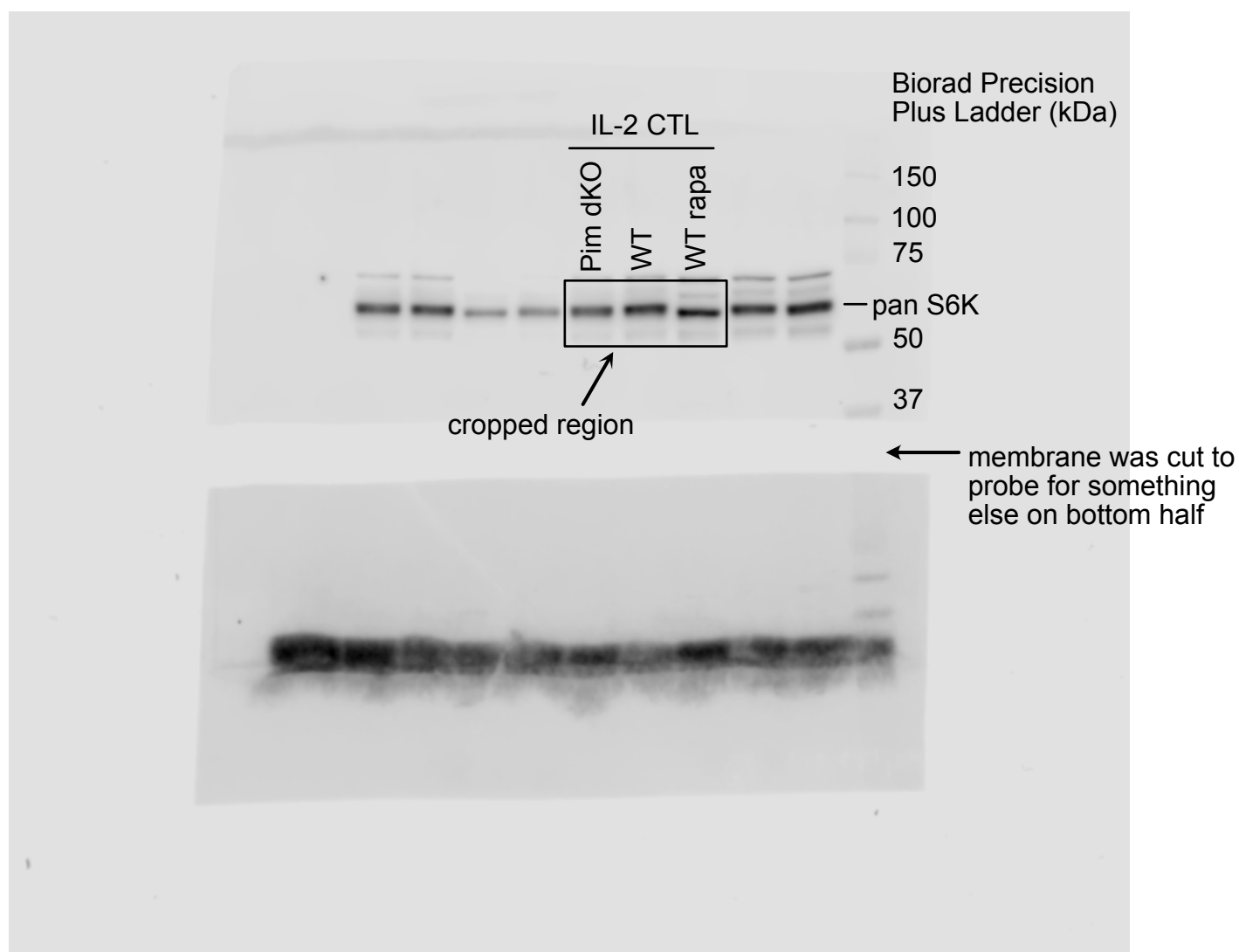

Figure 6 - source data 1

Uncropped and labelled membrane corresponding to Figure 6, panel A, pan S6K

Supplement: Figure 6—source data 1. [file elife-98622-fig6-data1.zip › Figure 6 - Source Data 1/Fig_6A_labelled_blot_pan_S6K.pdf]

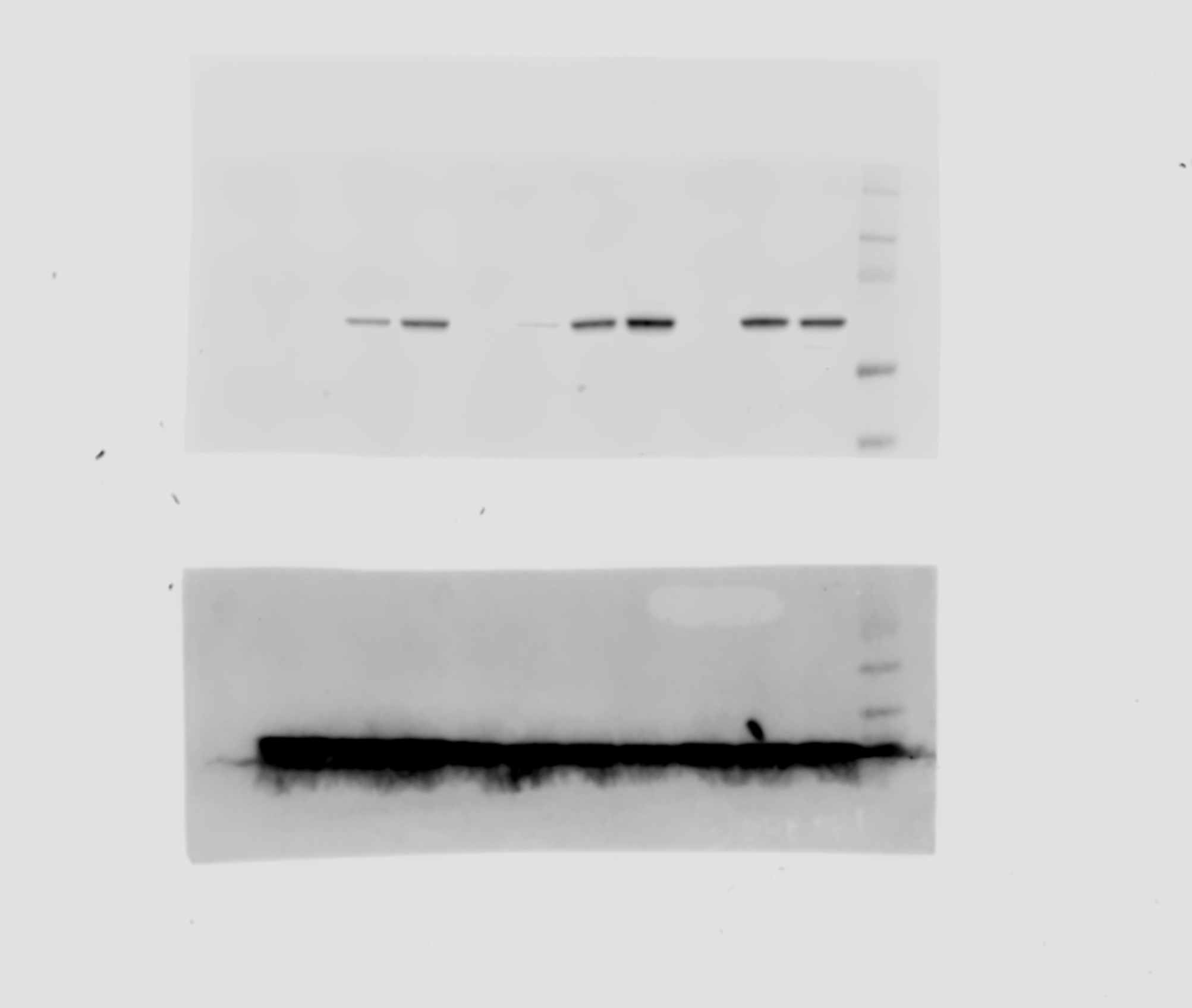

Supplement: Figure 6—source data 2. [file elife-98622-fig6-data2.zip › Figure 6 - Source Data 2/Fig_6A_raw_blot_phosphoS6K_T389.tif]

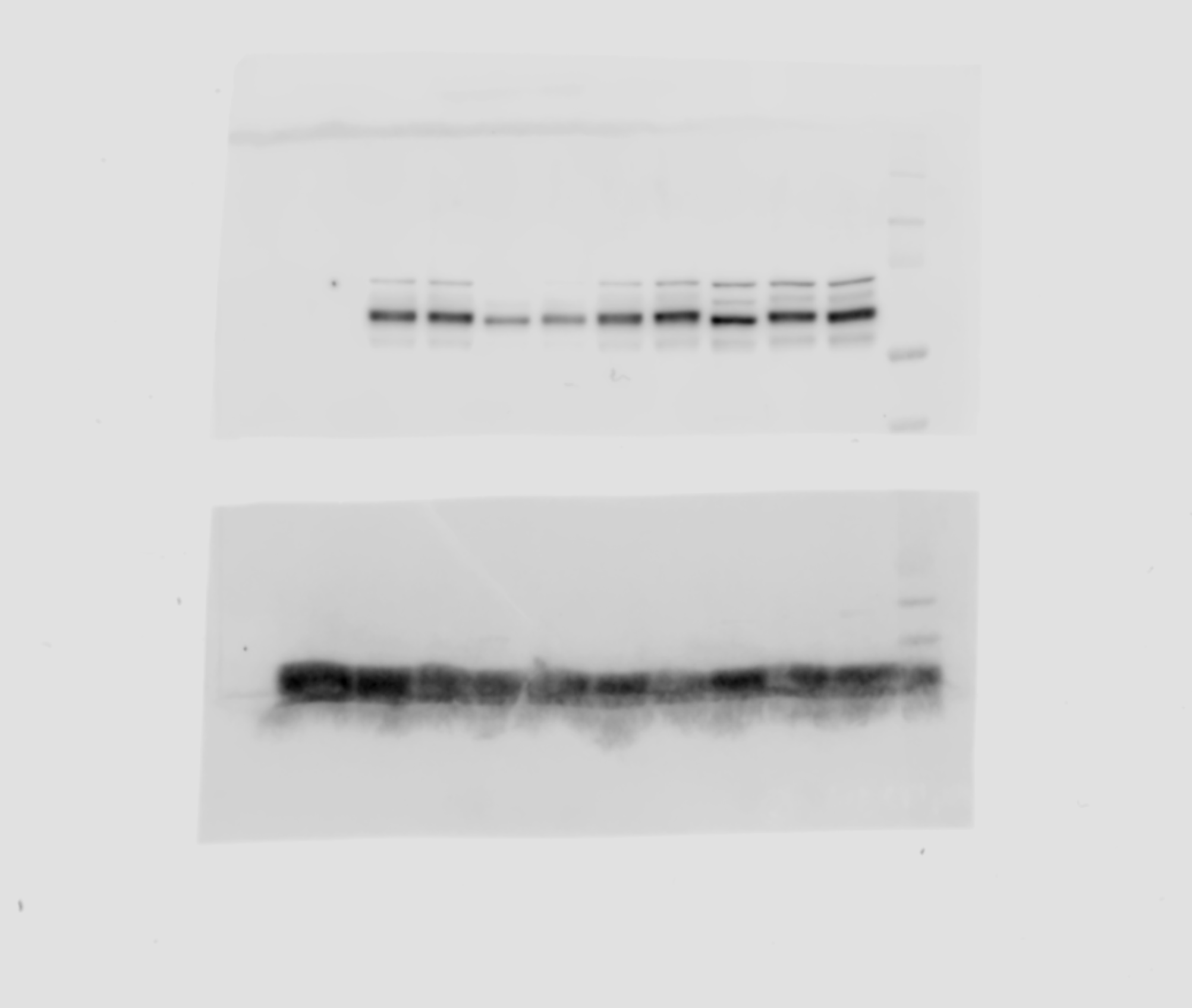

Supplement: Figure 6—source data 2. [file elife-98622-fig6-data2.zip › Figure 6 - Source Data 2/Fig_6A_raw_blot_panS6K.tif]
