# Supplementary material for: PIM kinase control of CD8 T cell protein synthesis and cell trafficking
Source: eLife. 2025 May 13;13:RP98622. doi: 10.7554/eLife.98622 (PMC12074636; doi:10.7554/eLife.98622)
Supplement: Figure 6—source data 1. [file elife-98622-fig6-data1.zip › Figure 6 - Source Data 1/Fig_6A_labelled_blot_phospho_S6K_T389.pdf]

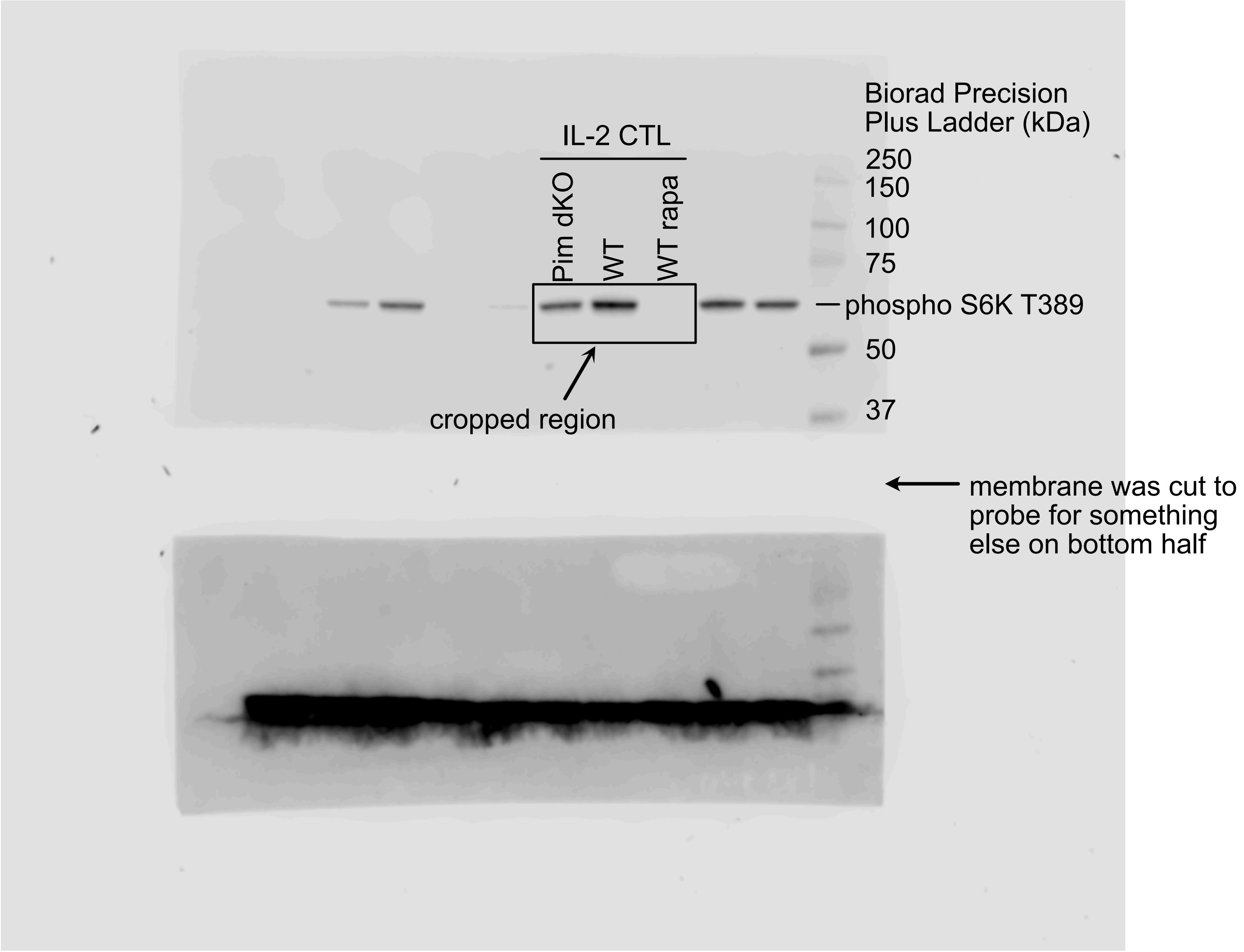

Figure 6 - source data 1

Uncropped and labelled membrane corresponding to Figure 6, panel A, phospho S6K T389
